# Annexins family: insights into their functions and potential role in pathogenesis of sarcoidosis

**DOI:** 10.1186/s12967-016-0843-7

**Published:** 2016-04-12

**Authors:** Mehdi Mirsaeidi, Sanaz Gidfar, Ann Vu, Dean Schraufnagel

**Affiliations:** Division of Pulmonary, Critical Care, Sleep and Allergy, Department of Medicine, University of Miami, Miller School of Medicine, 1600 NW 10th Ave # 7060A, Miami, FL 33136 USA; Department of Ophthalmology, University of Illinois at Chicago, Chicago, IL USA; Department of Medicine, University of Miami, Miami, FL USA; Division of Pulmonary and Critical Care, University of Illinois at Chicago, Chicago, IL USA

**Keywords:** Annexin, Sarcoidosis, Apoptosis, Calcium

## Abstract

Annexins are Ca^2+^-regulated phospholipid-binding proteins that play an important role in the cell life cycle, exocytosis, and apoptosis. Annexin A11 is one of the oldest vertebrate annexins that has a crucial role in sarcoidosis pathogenesis. The mechanism of effect in sarcoidosis granuloma cells may be due to alterations in apoptosis. Immune cells with a specific mutation at protein location 230 are resistant to apoptosis and consequently have continued effects on inflammation and progression of sarcoidosis. The mechanism of action of annexin A11 may be based upon alterations in delivering calcium to two different apoptosis pathways (caspase and P53).

## Background

Annexins (ANX) are a large family of calcium-dependent membrane-binding proteins. They are widely distributed among eukaryotes but largely absent in prokaryotes and yeasts. The first protein of this family, discovered in 1977, was called synexin. Currently known as annexin A7, it caused aggregation of chromaffin granules in the adrenal glands in the presence of free calcium [1, 2]. The medulla of bovine adrenal glands was incubated with various concentrations of synexin and chromaffin granules [1]. The regions of contact between granules formed by this protein were structurally similar to the contacts seen between vesicles and the plasma membrane in the process of exocytosis. Because of this ability to bring granules together, Creutz and colleagues used the term “synexin” from the Greek *synexis,* which means “meeting” [1]. The protein was found to be soluble, heat labile, and trypsin sensitive. Aggregation was also strongly temperature dependent. It did not cause granule aggregation in the presence of magnesium, barium, or strontium, but was activated by calcium [1]. Further characterization of these calcium and phospholipid binding proteins showed that they were localized to many different types of cells and tissues. The proteins of one group (calpactins) were substrates for tyrosine kinases. Because of the similar membrane-binding properties of these molecules and their conserved amino acid sequences, Geisow and colleagues called the group “annexins” [3]. Annexins were found to be receptors for calcium in exocytosis, promoting the close association of granules as well as the plasma membrane prior to secretion [4].

There is little information about annexin A11 and many of its structural and functional characteristics are speculated based on similarity with other members of annexin family. Recently, genetic mutation in annexin *A11* was linked to increasing susceptibility to sarcoidosis. It was also suggested that cells with mutant annexin *A11* might have altered susceptibility to apoptosis. However, the mechanism of this effect has never been discussed. We propose here a molecular mechanism for this association.

## Review

### Annexin family

More than a 100 annexins have been identified in many different species [5]. Twelve proteins have been identified in humans; these are conventionally referred to as annexin A1-13 (the ANX-A12 gene is unassigned). The descriptor ‘A’ denotes their presence in vertebrates; ‘B’ denotes their presence in invertebrates; ‘C’ denotes their presence in fungi and some groups of unicellular eukaryotes; ‘D’ denotes their presence in plants; and ‘E’ their presence in protists [5, 6]. The zebrafish demonstrates that the annexins are conserved through development. Zebrafish have eleven *annexin* genes [7] that are expressed in many tissues during embryonic and larval stages. Aligning the zebrafish *ANX* genes with mammalian *ANX* genes shows that three zebrafish *ANX* genes are homologous with human *ANX1*; two are homologous with human *ANX2*; and two are homologous with human *ANX11*. This information suggests that zebrafish *ANX* genes may have resulted from duplications after the divergence of the zebrafish and mammalian genomes [7].

### Human annexin genes

The 12 human annexin genes range in size from 15 kb (*ANXA9*) to 96 kb (*ANXA10*) and are spread throughout the genome on chromosomes 1, 2, 4, 5, 8, 9, 10, and 15 [6]. Other vertebral annexin genes may vary slightly in size and chromosomal linkage, but orthologues are similar in their sequence and splicing patterns. It is important that some annexin genes have been lost or duplicated in certain species, such as bony fish and pseudotetraploid frogs [8]. The duplication of annexin genes is also seen in humans [9]. Annexin A6 is a compound gene, probably derived from the fusion of duplicated *ANXA5* and *ANXA10* genes in early vertebrate evolution. The reasons for the annexin genes or their chromosomal regions to duplicate are not well understood. Their successful preservation and the extent to which they contribute to vertebrate complexity are also not well described [10]. The presence of multiple members of the annexin family in all higher eukaryotes argues for their fundamental role in cell biology.

The *ANXA11* gene is located on human chromosome 10q22**–**q23 and is composed of 15 exons and 14 introns without the 5′ flanking region [11]. Exon 1 is the biggest region of the gene and is untranslated. The N-terminal is coded by exons 2 through 5; exons 6–15 are responsible for the C-terminal.

**Fig. 1:**
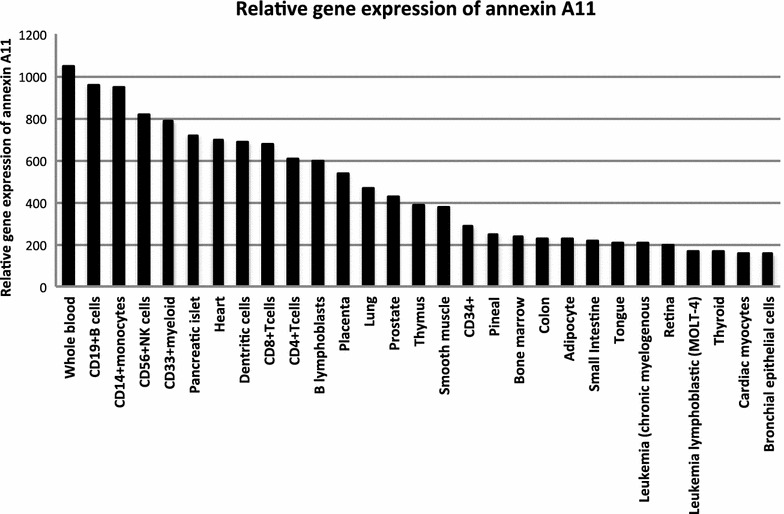
Tissue distribution of annexin A11 gene expression adapted from BioGPS website available at: http://biogps.org/#goto=genereport&id=311

Annexin gene expression levels within human organs have a broad range, from universal (for example, *annexins A1, A2, A4, A5, A6, A7, and A11*) to selective, such as annexin A3 in neutrophils, *annexin A8* in the placenta and skin, *annexin A9* in the tongue, *annexin A10* in the stomach and annexin A13 in the small intestine [6]. *ANXA11* has the highest gene expression in whole blood cells, particularly CD19^+^ cells (B-cells), CD14^+^ cells (monocytes) and CD33^+^ cells (myeloid). However, it is found in almost all tissues including lung, heart, and intestines [12]. Finding high expression of annexin A11 macrophages [13], neutrophils [14] and T-cells [15] suggests it may have a significant role in immune system function and possibly in a number of autoimmune diseases (Fig. 1).

High expression patterns of annexins are detected in thymus, lung, and smooth muscle, and low expression patterns are found in testis, adrenal glands, and brain. The expression of annexins may change with the cell cycle. For instance, while the cell cycle progresses, the distribution of annexin A11 changes. This may be because it is associated with microtubules, vesicle trafficking, and Ca^2+^ regulated exocytosis [1, 16, 17].

### Protein structures

All annexins share a conserved C-terminal core domain made up of at least four similar repeats, each about 70 amino acids long [18]. These subunits usually contain characteristic ‘type 2′ calcium binding sites. The number and location of these sites generally vary between different annexin families, with variation and replacement with other motifs [5, 19]. Calcium-independent annexin membrane interactions involve a switch from a helix-loop-helix motif to the transmembrane helix, which drives a reversible membrane insertion. This pH-dependent conformation switch can be induced by the protonation of certain carboxylate residues found close to the loop of the helix-loop-helix motif. This model may explain why annexins can span a lipid bilayer [5]. In contrast to the core domain, individual vertebrate annexins have a unique N-terminal domain of variable length, amino acid sequences, and determinants of hydrophobicity. This plays an important role in mediating the interaction of annexins with other intracellular protein partners, such as those of the S100 family cytoplasmic proteins [20]. The NH2-terminal domain of annexin A2 forms a protein-protein interaction through a highly specific binding site for the small dimeric S100 protein S100A10 [21]. This heterotetrameric complex is formed when two annexin A2 molecules are non-covalently linked via a S100A10 dimer bound to their NH2-terminal domains. As a result, this complex can bind simultaneously to two membrane surfaces through its two annexin A2 cores [5].

Nonhuman annexin protein structure has also been studied. The simplest organisms known to express annexins are the protist *Giardia lamblia* and the fungus *Neurospora crassa.* The structure of nonhuman annexin protein was discussed elsewhere [22, 23]. Plant annexins have a structure that is distinct from vertebrates. They lack the variable N-terminal domains and type II calcium binding sites [24].

**Fig. 2:**
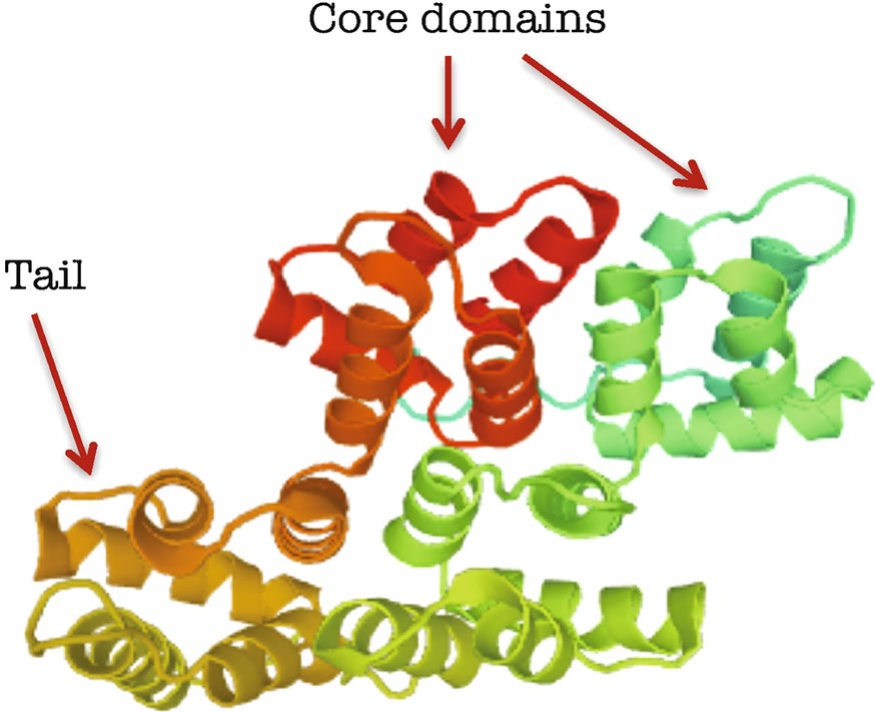
Model of the annexin A11 secondary structure with four core domains and a rich tail

ANXA11 contains 504 amino acids and has a molecular weight of 56 kDa [25]. Its primary structure was purified from rabbit lung in 1992 [26]. ANXA11 has a C-terminal core and a N-terminal head with 50 % homology with other annexin core domains [27]. The C-terminal core contains four domains with calcium binding properties (Fig. 2). Although ANXA11 has not been crystallized yet, its tertiary structure was predicted by information from ANXA1 and ANXA5 [28]. Three isoforms of Annexin A11 are identified in humans, but only one is expressed in cells [29].

### Intracellular location and function of annexins

Annexins are usually cytosolic proteins with a soluble and a stable form. Annexins are reversibly associated with components of the cytoskeleton or proteins that mediate interactions between the cell and the extracellular matrix (matri-cellular proteins). Some annexins, such as annexins A11 and A2, have been found in the nucleus under particular circumstances in the cell cycle [16, 17]. It seems that annexin A2 works with annexin A11 in the nucleus. When annexin A2 enters the nucleus, it is rapidly exported to a functional nuclear export (NES) sequence that overlaps the p11-binding region in the annexin 11 N-terminus [21]. When p11 binds to annexin 11, the complex is sequestered in the cytoplasmic compartment [16, 21].

In some circumstances, annexins can be expressed at the cell surface even without a secretory signal peptide. For example, annexin A1 translocates from the cytosol to the cell surface following exposure of cells to glucocorticoids [30]. In a study involving U-937 cells, annexin 1 translocated from the intracellcular compartment to the cell membrane without a signal exporting sequence. The level of expression of annexin 1 was directly related to the time exposed to dexamethasone. Prior to its release, the protein may accumulate in the cell membrane, and this is stimulated by dexamethasone in differentiated U-937 cells [30].

Annexin A2 expression at surface of vascular endothelial cells has a function in the regulation of plasmin generation [31]. It has been shown that Annexin A2 is a co-receptor for tissue plasminogen activator and plasminogen. This expression can be found on a variety of cells, including endothelial cells, tumor cells, and macrophages. This study also showed that Annexin A2 may also have a role in maintaining vascular patency and the cell formation of new blood vessels [31].

Annexin A11 distribution changes per cell cycle. It is more prevalent in the nucleus than the cytoplasm in interphase cells and then moves to degenerating nuclear envelope. And finally during the mitotic phase, it will be concentrated within the central spindle [16]. It has been shown that Annexin A11 also has an essential role in the terminal phase of cytokinesis. Annexin A11 is recruited to the midbody in late telophase, and without Annexin A11, cells cannot establish a functional midbody. [32]. Instead, daughter cells remain connected by intercellular bridges. As a result, these cells without Annexin A11 do not complete cytokinesis and die by apoptosis [32].

### Annexins interaction with other proteins

S100 proteins, which only express in vertebrates, are a well-known group of proteins that interact with the annexins. The functions of S100 proteins are diverse and include regulating actin and microtubule networks, promoting cell survival and proliferation, calcium homeostasis, and mediating muscle contraction. The complex of annexin A2-S100A10 interacts with several membrane ion channels, such as transient receptor like potential vanilloid type 5 and 6 channels (TRPV5 and TRPV6). The complex also interacts with cystic fibrosis conductance regulator protein (CFTR) and plays a role the regulation of these ion channels [33, 34]. It is clear that these proteins are involved in a great number of intracellular processes, such as membrane trafficking, organization, and functioning as extracellular local hormones.

Annexin A11 is involved in cellular apoptotic processes. The N-terminal domain of ANXA11 contains binding sites that deliver Ca^2+^ to S100A6 and apoptosis-linked gene2 (ALG-2) whose protein augments apoptosis. The significance of these interactions in the pathogenesis of sarcoidosis is discussed later in this article.

### Association of annexin to diseases in laboratory animal models

Studies on knockout mice of annexin families show a diversity of functions among these proteins. Loss of *ANXA1* causes changes in the inflammatory response and the effects of glucocorticoids [35]. In the *ANXA1* null mouse line, there was altered expression of other annexins as well as cyclooxygenase-2 and cytoplasmic phospholipase A2. In addition, there was an exaggerated response to the stimuli characterized by an increase in leukocyte emigration and IL-1β generation and a partial or complete resistance to the anti-inflammatory effects of glucocorticoids [35].

Data supports the role of annexin 2 as a regulator of cell surface plasmin generation, fibrin homeostasis, and neovascularization in laboratory mice models [36]. Homozygous annexin 2 knockout mice were studied, and they showed deposition of fibrin in the microvasculature. These null mice also had deficits in the clearance of arterial thrombi and tissue plasminogen activator (T-PA)–dependent plasmin generation at the endothelial cell surface. Also, annexin 2–deficient mice displayed problems with neovascularization of fibroblast growth factor–stimulated cornea and of oxygen-primed neonatal retina [36].

Another study involved the formation of the *ANXA7* knockout mouse. The viability of the *ANXA7* null mouse was compared to the heterozygous mouse [37]. The ANXA7 null mutation mouse was did not survive past embryonic day 10. This was due to cerebral hemorrhage. On the other hand, the heterozygous mouse, though only expressing low levels of *ANXA7*, was viable and able to reproduce [37].

Another study of *ANXA7* null mutant mouse proposed *ANXA7* function in the fusion of vesicles as a calcium channel [38]. Cardiomyocytes from adult *ANXA7* null mice were studied. When stimulated with high frequencies, the cells showed an altered cell shortening relationship. Possibly through its role in calcium regulation, this study suggested a function for annexin A7 in electromechanical coupling [38]. The other annexins knockouts need further investigation.

### Association of annexin to human diseases

Annexins have essential roles in the pathogenesis or progression of many human diseases. Recent genetic studies discovered single nucleotide polymorphisms (SNPs) in the genomes of this group of proteins. In a study from India, annexin A2 gene SNP (rs7170178) was found to be associated with osteonecrosis in sickle cell patients [39]. The frequency of the *ANXA2* gene polymorphism was higher in the sickle cell patients compared to controls. The SNP was also present in higher frequency in sickle cell osteonecrosis patients than those without osteonecrosis [39].

In another study from Japan, the annexin A5 gene polymorphism was found to be associated with recurrent pregnancy loss. The promoter region of the *ANXA5* gene was sequenced in 243 Japanese women with recurrent pregnancy loss and 119 fertile controls [40]. In a case control study for six common *ANXA5* gene SNPs, the carrier frequency for the minor allele was significantly higher in the pregnancy loss group [40]. For SNP5, women with this minor allele had a two-fold higher risk of fetal loss than non- carriers. Homozygotes for the SNP5 minor allele had a seven-fold higher risk of recurrent pregnancy loss [40].

Annexins are also associated with autoimmune disorders. In rheumatoid arthritis, high extracellular annexin V levels initiates the production of annexin V autoantibodies that may have a crucial role in pathogenesis of disease [41]. Systemic lupus may involve defective clearance of dying cells, resulting in the exposure of nuclear antigens in the form of cellular debris or microparticles. These microparticles may contain antigens that trigger autoimmune processes. Lupus patients have decreased annexin V binding microparticles and an increase in annexin V non-binding microparticles [42].

Dysregulation of Annexin A11 has been found in cancer, cancer treatment, and diabetes [43]. For example, Annexin A11 is directly involved in cell proliferation in ovarian cancer [44]. The knockdown of annexin A11 expression reduced cell proliferation and the ability of ovarian cancer cells to form a colony. Silencing of annexin A11 was also associated with cisplatin resistance in ovarian cancer cells. [44, 45]. ANXA2 is also involved in P53-mediated apoptosis of lung cancer cells [46]. It has been shown that drug-resistant small cell lung cancer cells highly express annexin A2. Thus, Annexin A2 may have a role in pathogenicity of drug resistance [47].

Some members of the annexin family may also be used as biomarkers and for clinical imaging. For example, ANXA1 was investigated as a potential serum biomarker for lung cancer. Lung cancer tissues exhibited higher expression of annexin A1 than normal tissues. In addition, increased serum annexin A1 was significantly associated with pathologic grade and clinical stage of lung cancer patients [48]. Quantitative 99 mTc-annexin A5 (qAnx5) imaging uses human annexin A5 radiolabeled for the visualization and measurement of apoptosis. This imaging is being investigated as an objective evaluation of apoptosis before and after cancer treatment. Annexin A5 may be used as clinical imaging marker for treatment response [49].

### ANXA11 and sarcoidosis

Sarcoidosis is a systemic immune disorder with a characteristic accumulation of epithelioid granulomas in many organs, such as the lungs, kidney, skin and eyes [50, 51]. Chronic sarcoidosis is disease activity lasting more than 2 years [52, 53]. One of the consequences of chronic sarcoidosis is pulmonary fibrosis [54]. Pulmonary fibrosis occurs in 20 % of patients and contributes significantly to morbidity and mortality among these patients [55–58].

**Fig. 3:**
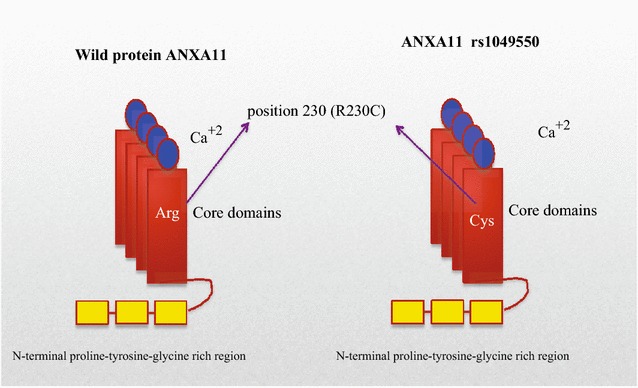
ANXA11 polymorphism in location 230. Created based on this article: Alejandra Tomas and Stephen E Moss J Biol Chem 2003, 278:20210–20216

**Fig. 4:**
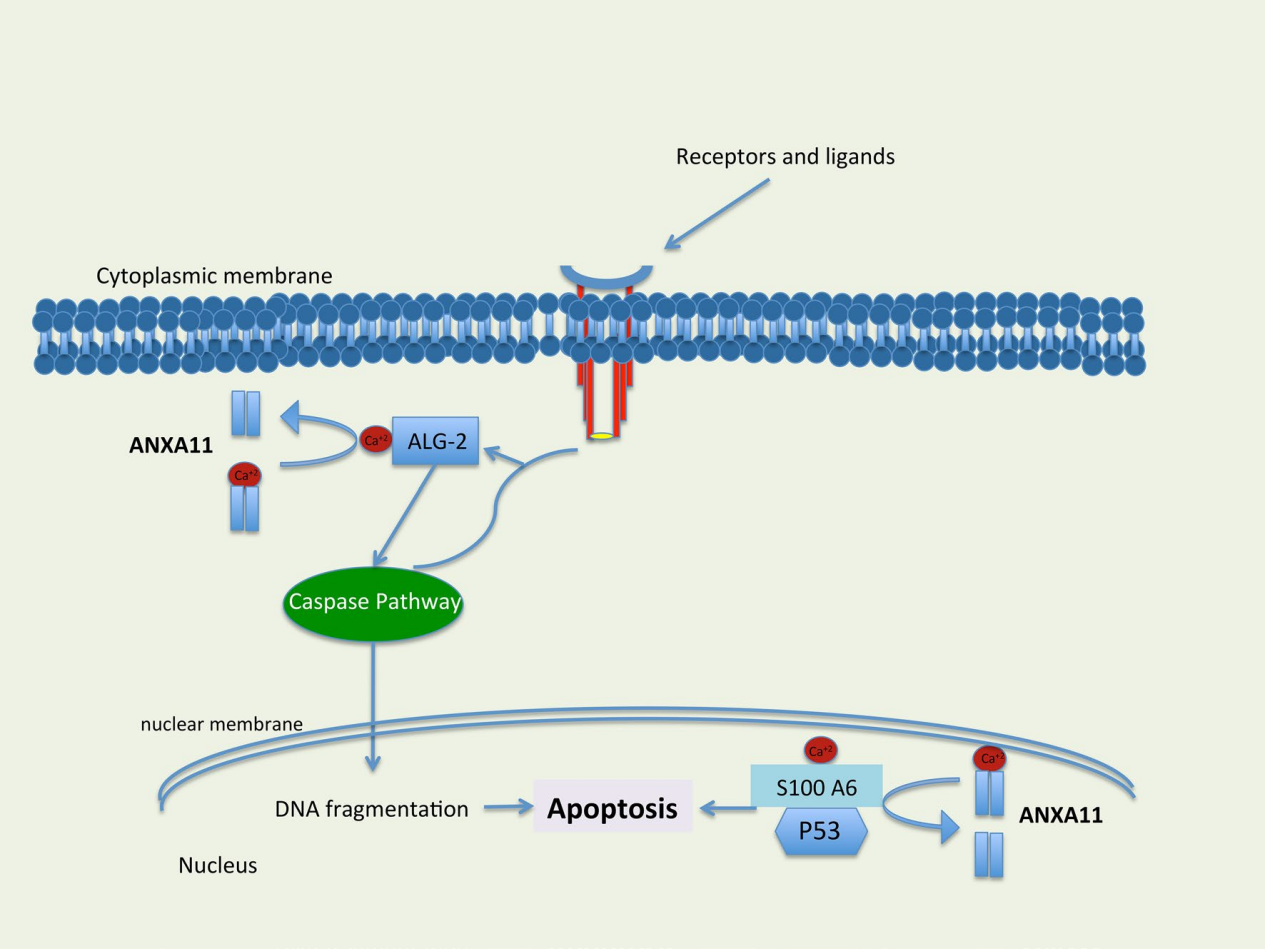
The role of ANXA11 in apoptosis. ANXA11 with delivering calcium (ca^2+^) to ALG-2 in caspase pathway and s100A6 in P53 pathway promote apoptosis. Reproduced based on Satoh and coworkers paper [61]

Genetic instability and mutation in annexin A11 has been identified in single nucleotide polymorphisms in patients with sarcoidosis compared to control groups. Decreased activation of CD8^+^ and CD19^+^, immune cells involved in sarcoidosis, are proposed mechanisms for sarcoidosis [15]. In addition to the SNP discovery, sarcoidosis patients show an increase in neutrophil counts in bronchoalveolar lavage fluid. This has led to the investigation of annexin A11, which is important in cell division, apoptosis, and neutrophil function. In a study of more than 440,000 SNPs of 490 German patients with sarcoidosis, a series of genetic associations were detected compared with controls [15]. The strongest association signal maps to the *ANXA11* (annexin A11) gene on chromosome 10q22.3. A common nonsynonymous SNP (rs104955) was found to be strongly associated with sarcoidosis. As it is demonstrated in Fig. 3, this SNP causes a substitution of arginine with cysteine at position 230 (R230C). Although the mechanistic effect of this change has not been well defined, it appears to affect apoptosis and proliferation in sarcoidosis [15]. Fillerova and coworkers showed that peripheral blood mononuclear cell (PBMC) isolated from subjects with sarcoidosis who carried the *ANXA11* R230C SNP were more resistant to apoptosis than the wild genotype. This association was particularly prominent in subjects with the TT *ANXA11* phenotype [59]. The mechanism of this increasing resistance to apoptosis was not discussed. We theorize that ANXA11 with structural changes after SNP R230C loses all or part of its functionality. As mentioned above, ANXA11 carries 4 calcium ions and delivers calcium to many intracellular pathways. ANXA11 is involved in apoptosis in at least two known pathways. As shown in Fig. 4, ANXA11 is involved in mitogen-activated protein kinase (MAPK) and P53 pathways. Mitogen-activated protein kinase pathways are involved in apoptosis in the setting of environmental stress [60]. The MAPK pathway activates caspase pathway via an ALG-2 protein that is Ca^2+^ dependent. Without calcium delivery from ANXA11 to ALG-2, the apoptosis via caspase pathway would not be activated [61].

**Fig. 5:**
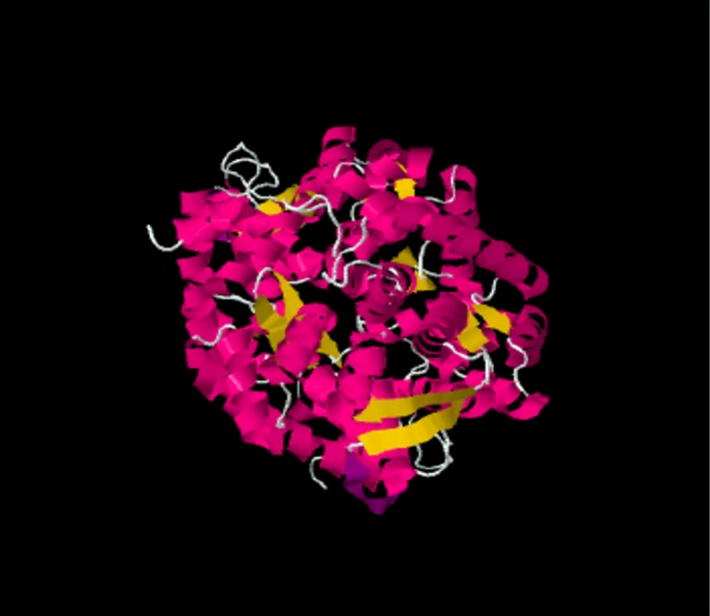
The 3rd structure of disulfide bonds in ANXA11. Modeled using Disulfide Bonds in Proteins (MODIP, http://caps.ncbs.res.in/dsdbase/modip.html) and Disulfide by design (DbD, http://cptweb.cpt.wayne.edu/DbD2/)

The second important protein involved in apoptosis is P53 [62]. P53 mediated apoptosis pathway starts with joining S100 A6 to P53 which needs Ca^2+^ delivery from ANXA11 [16]. Indeed, if ANXA11 R230C could not deliver calcium to ALG-2 and S100A6, the apoptosis would not occur. However, how could the substitution of arginine with cysteine make the protein dysfunctional? The possible mechanism could involve the disulfide bond between the two cysteine thiol groups. One group is native with a close distance to another non-native group, which comes from R230C. The two groups form a disulfide bridge. Disulfide bonds provide a mixed outcome, either an increase or decrease in protein structure stability [63]. With a new cysteine in the structure of ANXA11 R230C, a non-native disulfide bridge is formed. This misfolds the protein and renders it nonfunctioning [64]. Figure 5 shows the third structures of disulfide bonds in ANXA11.

**Fig. 6:**
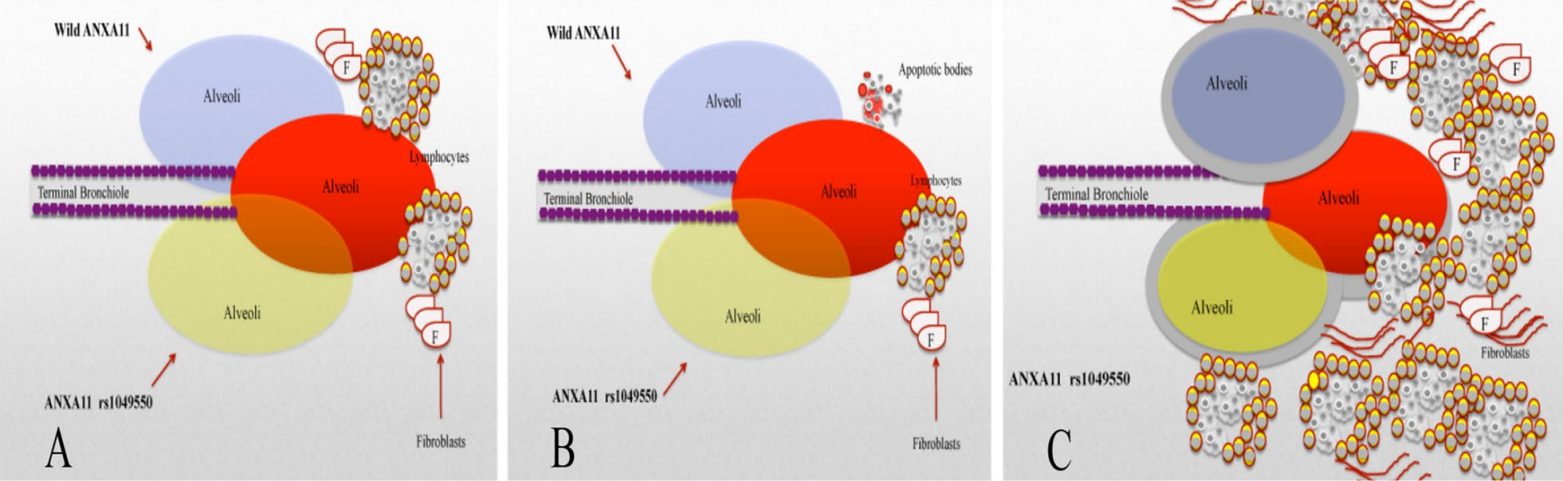
Demonstrates our proposed mechanism of effect of *ANXA11* rs1049550 in progression of sarcoidosis. **a** Shows onset of granuloma in wild type (*above*) and mutant type of ANXA11, **b** Shows regression of granulomatous reaction with apoptosis in wild type *ANXA11* (*above*) and continuation of granuloma with resistant to apoptosis in mutant type of *ANXA11* (*below*). **c** Shows continuation of granuloma cause fibrosis changes in the mutant type of *ANXA11*

In the sarcoidosis lung, granulomas develop when the immune cells attempt to wall off an unknown antigen trigger. These cells include macrophages, lymphocytes, and multinucleated giant cells. The granuloma can be effaced by apoptosis leaving only minor scar. Without apoptosis the inflammatory and granulomatous reaction continues. Patients with ANXA11 R230C cannot clear granulomas due to altered apoptosis and may have a higher risk of onset of sarcoidosis and poorer outcome (Fig. 6).

### Pre-clinical advances in annexin and sarcoidosis

Hofmann et al. were the first group of researchers to identify the association of ANXA11 with sarcoidosis [15]. More recent studies have confirmed that Annexin A11 and its regulation of apoptosis is a key genetic player in the development of sarcoidosis. The latest in sarcoidosis genetics research uses genome mapping to determine gene variants associated with increased granuloma formation [65]. A 2014 study of the Han Chinese population confirmed the potential role of ANXA11 SNPs in the genetic susceptibility to sarcoidosis. Investigators studied 15 potential loci and found significant differences between patients with sarcoidosis and healthy controls for 3 SNPs. They showed that rs1049550 presented a significant effect on disease phenotype (p < 0.001). The T allele was an important protective factor against sarcoidosis. Whereas carriers of rs1049550, the C allele, had a higher susceptibility to sarcoidosis. This was confirmed in association analysis, which showed that the T–C haplotype occurred significantly less frequently and the C–C haplotypes occurred more frequently in in patients with sarcoidosis [66].

In a 2013 study, the role of annexin A11 in sarcoidosis was confirmed in a US population. This association also extended to clinical manifestation of disease. The studied involved 1689 sarcoidosis cases and 1252 controls. 25 SNPs in and around ANXA11 were significantly associated with sarcoidosis risk in both African Americans and European Americans. The most significant allelic association was the minor C allele at rs1049550 among African and European Americans [67]. This was the same allele identified in the original study by Hofmann et al. [15]. ANXA11 mutations also conferred risk for radiographic expression of disease. For SNP rs4399277, the strongest association was found in cases with Scadding stage IV disease. The A allele was associated with a 1.52-fold increase in the odds of stage IV disease. New independent loci associated with sarcoidosis risk in ANXA11 were also identified. The minor A allele of SNP rs61860052 was associated with a protective effect on sarcoidosis in African Americans [67].

These recent advances of annexin A11 in the pre-clinical stage show its significant role in the pathogenesis of sarcoidosis. In the era of personalized medicine, the annexin could be a novel focus. Annexin A11 thus may be a target for clinical trials in which a patient’s genetic risk factors are used to tailor treatment [65].

## Conclusions

Annexins have significant effects on human health and disease. Annexin A11 affects two apoptosis related pathways (caspase and P53) and has a crucial role in sarcoidosis pathogenesis. This theory should be tested in cell culture and animal models. Recent studies show the significant role of annexin A11 in the genetic susceptibility to sarcoidosis. If the ANXA11 could increase susceptibility to sarcoiodosis via interaction with caspase and P53, a new opportunity to develop new therapeutic targets in sarcoidosis will be recognized.

## Abbreviations

ANX: annexins
NES: nuclear export
TRPV5 and TRPV6: transient receptor like potential vanilloid type 5 and 6 channels
CFTR: cystic fibrosis conductance regulator protein
ALG-2: apoptosis-linked gene2
T-PA: tissue plasminogen activator
SNPs: single nucleotide polymorphisms
PBMC: peripheral blood mononuclear cell
MAPK: mitogen-activated protein kinase
